# Elevated levels of TRAb IgG autoantibodies are not recognized in endometriosis by the current clinical methods

**DOI:** 10.3389/fmed.2025.1612079

**Published:** 2025-09-22

**Authors:** Agnes Petersson, Bodil Roth, Charlotte Becker, Bodil Ohlsson

**Affiliations:** ^1^Depertment of Clinical Sciences, Lund University, Malmö, Sweden; ^2^Department of Internal Medicine, Skåne University Hospital, Malmö, Sweden; ^3^Department of Clinical Chemistry and Pharmacology, University Laboratory, Skåne, Sweden

**Keywords:** endometriosis, irritable bowel syndrome (IBS), TSH receptor antibodies (TRAb), autoantibody, biomarker

## Abstract

**Background:**

Endometriosis can cause gastrointestinal symptoms that sometimes lead to incorrect diagnosis of irritable bowel syndrome (IBS). Receptors of thyroid-stimulating hormone (TSH) are found in the endometrium and are overexpressed in ectopic endometrium. The role of TSH receptors and thyroid hormones in the pathophysiology of endometriosis has therefore been discussed. No biomarker is available for endometriosis diagnosis, but the findings of TSH receptor antibodies (TRAb) have been found by two different methods. The present study aimed to confirm that TRAb IgG levels are elevated in patients with endometriosis compared with IBS and healthy controls.

**Methods:**

A total of 121 patients with a diagnosis of endometriosis and two cohorts with 50 IBS patients and 50 healthy controls were recruited for the study. All subjects had to fulfill study questionnaires regarding sociodemographic factors, lifestyle, medical history, and gastrointestinal symptoms. Blood samples were drawn, and TRAb IgG was analyzed in serum samples at the Departments of Clinical Chemistry at Sahlgrenska University Hospital using the BRAHMS TRAK Human assay from Thermo Fisher Scientific and at Skåne University Hospital using the Elecsys Anti-TSHR assay from Roche Diagnostics. Both methods were developed and used clinically to diagnose Graves’ disease with high specificity.

**Results:**

Patients with endometriosis had the highest BMI (*p* = 0.001) and prevalence of hypothyroidism (*p* = 0.005). Endometriosis and IBS patients had many gastrointestinal symptoms, in contrast to the healthy controls. There was no significant difference in the number of patients with detectable serum TRAb between endometriosis patients (*n* = 10, 8.3%) and controls (*n* = 2, 4%) (*p* = 0.512) or between endometriosis patients (*n* = 10, 8.3%) and IBS patients (*n* = 3, 6%) (*p* = 0.758) in Gothenburg. Similar results were found when TRAb was analyzed in serum samples in Malmö, with the same prevalence of TRAb in endometriosis patients (*n* = 4, 4.9%) as in controls (*n* = 4, 8.0%) (*p* = 0.710) and IBS patients (*n* = 4, 8.0%) (*p* = 0.710).

**Conclusion:**

The available tests in clinical routine could not reveal elevated levels of TRAb in the current exploratory study. Thus, TRAb cannot yet be used clinically as a biomarker of endometriosis. Still, other variants of antibody tests may be used in a laboratory experimental setting. The role of TSH receptors and TRAb in the pathophysiology of endometriosis deserves further research.

## Introduction

Endometriosis is an inflammatory disease characterized by the localization of endometrium outside the uterus (1). Estrogen is deeply involved in the pathophysiology of endometriosis since it generates local and systemic inflammation, which enables the transplantation of endometrial tissue outside the uterus and affects proliferation of the cells (2, 3). There is a strong interaction between estrogen and thyroid hormones through the hypothalamic–pituitary–thyroid axis (4). The human endometrium expresses mRNA for thyroid-stimulating hormone (TSH) receptors and thyroid hormone receptors (5, 6), which produce thyroid hormones in response to TSH, thereby functioning as a site for extra-thyroidal hormone production (6). TSH receptors are overexpressed in ectopic endometrium, and thyroxine (T4) has a specific proliferative effect on ectopic endometrial cells (7).

Some authors have found Hashimoto’s disease to be associated with endometriosis (8), whereas others have found an association between Graves’ disease and endometriosis (9). However, no association could be found between endometriosis and thyroid disorders in the general population. Notably, endometriosis patients with concomitant thyroid dysfunction had more chronic pelvic pain than euthyroid patients (7). This may result in patients with both endometriosis and thyroid disorders seeking medical healthcare more frequently and therefore being more likely to participate in research studies..

Three previous studies have reported elevated TSH receptor IgG antibodies (TRAb) in patients with endometriosis compared to controls from the general population (10–12). TRAb IgM has been shown to be elevated in endometriosis compared with healthy blood donors (11). Contrasting results have also been reported, where no association between TRAb and endometriosis was identified (13). Other studies have found a higher prevalence of elevated levels of peroxidase antibody (TPO-Ab) in endometriosis (14), which could not be confirmed in a later study (10). Antibodies against other members of the glycoprotein hormone/glycoprotein hormone receptor family were, however, not increased, indicating that the findings of TRAb were not caused by cross-reactivity of this family (11).

Due to the abovementioned results, there is an ongoing debate concerning the relation between thyroid immunity and the pathophysiology of endometriosis. Currently, no biomarkers are approved in clinical practice to support the diagnosis of endometriosis. This often leads to a long delay before the endometriosis diagnosis is set (15), and many patients with gastrointestinal symptoms caused by endometriosis are incorrectly diagnosed as suffering from irritable bowel syndrome (IBS) (16, 17). The present study aimed to confirm, in new cohorts and in a different laboratory setup, that the levels of TRAb IgG are truly elevated in patients with endometriosis compared to patients with IBS and healthy controls.

## Methods

### Study design

This is a cross-sectional study comparing endometriosis patients (*n* = 121) with two cohorts of IBS patients (*n* = 50) and healthy controls (*n* = 50). All study participants answered a study questionnaire concerning sociodemographic factors, lifestyle habits, medical history, and the visual analog scale for irritable bowel syndrome (VAS-IBS). IBS patients and controls completed the irritable bowel syndrome-severity scoring system (IBS-SSS), and IBS patients also completed the Rome IV questionnaire. Blood samples were collected, and the body mass index (BMI) was calculated from weight and height. Serum concentrations of TRAb IgG in endometriosis and IBS patients and healthy controls were analyzed according to clinical routines in 2023 at the Department of Clinical Chemistry at Sahlgrenska University Hospital, Gothenburg. Since the results were negative, with few detectable antibodies, the samples were also analyzed at the Department of Clinical Chemistry at Skåne University Hospital, Malmö, Sweden.

### Patients

Two cohorts of patients with endometriosis were included in the study. The first cohort consisted of patients with endometriosis confirmed by laparoscopy or laparotomy. They were recruited from the Department of Gynecology, Skåne University Hospital, Malmö, during the periods 2013–2014 and 2016–2017, after a search in the medical records according to the International Statistical Classification of Disease and Related Health Problems—ICD-10 code of endometriosis (N80). The recruitment process and basal characteristics are described in a previous publication (10). Briefly, 605 patients fulfilling the inclusion criteria were identified. Of those, 32 had significant comorbidity, 307 declined to participate, 72 had moved, and 22 had an uncertain diagnosis or denied the diagnosis. In total, 172 patients were included. Those patients who had not previously been analyzed for TRAb IgG at a detection limit of 0.3 IU/L (n = 40) were selected for analysis in Gothenburg. The second cohort was recruited between 2022 and 2023 from the same department. Patients who were examined by transvaginal ultrasound, according to the new guidelines for diagnosing endometriosis (18), were asked to participate in the study if the diagnosis was verified. In total, 96 women were considered to participate, of which 81 accepted the invitation. Serum from these 81 women was analyzed in both Gothenburg and Malmö. The inclusion criteria for both cohorts were patients aged 18–70 years, those with a diagnosis of endometriosis, and those with the ability to comprehend the Swedish or English language. The exclusion criteria were multiple or severe somatic or psychiatric comorbidities and current pregnancy. Localization of endometrial lesions was categorized into isolated ovarian endometriosis or spread endometriosis.

### IBS

Patients with IBS were originally recruited at the Department of Internal Medicine, Skåne University Hospital, Malmö, for a dietary trial between December 2021 and September 2023, as previously described in detail (19). The inclusion criteria were patients aged 18–70 years and those with a diagnosis of IBS according to Rome IV with a total IBS-SSS > 175 (20, 21). The exclusion criteria were multiple or severe somatic or psychiatric comorbidities and current pregnancy. Patients who had received a diagnosis of IBS according to the ICD-10 codes K58.1 (diarrhea-predominated IBS; IBS-D), K58.2 (constipation-predominated IBS; IBS-C), K58.3 (mixed IBS; IBS-M), and K58.9 (other and unspecified IBS; IBS-U) were identified from medical records of the County of Skåne, referrals from healthcare centers, or advertisements on social media. Of the 300 subjects, 214 were randomized to participate in the study, of which 155 patients with IBS were finally included in the dietary trial. Of these 155 patients, 50 women were randomly selected for analysis of TRAb in Gothenburg and 50 were selected for analysis in Malmö. A total of 24 women were analyzed in both Gothenburg and Malmö.

### Controls

A control group consisting of healthcare workers and medical students from Skåne University Hospital, Malmö, aged 18–70 years, was recruited through personal invitations and advertisements. The exclusion criteria were chronic or acute illness or having significant gastrointestinal symptoms (22). In total, 74 healthy controls were recruited. Of these 74 controls, 50 women were randomly selected for the analysis of TRAb in this study.

### Questionnaires

All study participants completed a previously developed study questionnaire regarding sociodemographic factors, lifestyle habits, medical history, and pharmacological treatment (10). The VAS-IBS was used to estimate gastrointestinal symptoms, measuring abdominal pain, diarrhea, constipation, bloating and flatulence, vomiting and nausea, psychological wellbeing, and intestinal symptoms’ influence on daily life on scales from 0 to 100 mm, where 0 represents no symptoms and 100 represents severe symptoms. The scales were inverted from the original format (23). Reference values are available from healthy women (24). IBS patients and healthy controls also completed the IBS-SSS regarding abdominal pain, abdominal distension, satisfaction with bowel habits, and the impact of bowel habits on daily life, which estimated the symptoms using visual analog scales (VAS) ranging from 0 mm to 100 mm, and days with abdominal pain in the last 10 days were reported to assure they fulfilled the inclusion and exclusion criteria. The maximum achievable score is 500. Scores <75 indicate absence of disease, scores ranging 75–174 indicate mild disease, score ranging 175–299 indicate moderate disease, and scores ≥300 indicate severe disease (21). The IBS patients completed the Rome IV questionnaire, questions 40–48 in the Swedish version, to confirm the IBS diagnosis. The license was obtained from the Rome Foundation, Inc., Raleigh, NC, USA (25).

### TRAb analyses

Analysis of IgG levels of TRAb in serum samples was performed according to the standardized methods at the Departments of Clinical Chemistry in Gothenburg and Malmö. Analyses were performed in 2023. The Department of Clinical Chemistry at Sahlgrenska University Hospital in Gothenburg analyzes TRAb using the BRAHMS TRAK HUMAN KRYPTOR assay on a Kryptor compact PLUS from Thermo Scientific, Hennigsdorf, Germany. The analysis is based on a monoclonal competitive TRACE technique (time-resolved amplified cryptate emission). The lowest detection limit was 0.26 IU/L, and the limit of quantification was 0.93 IU/L (defined as the level where the coefficient of variation (CV) is <20%). Concentrations of TRAb <1.4 IU/L were considered negative, concentrations 1.4–1.8 IU/L were considered gray zone, and concentrations >1.8 IU/L were considered positive for Graves’ disease. Intra-assay CV% was 12 at concentrations 1–1.2 IU/L (26).

The Department of Clinical Chemistry in Malmö analyzes TRAb with the Elecsys Anti-TSHR assay on a cobas® pro from Roche Diagnostics, Mannheim, Germany. The analysis is a monoclonal competitive method with the Electrochemiluminiscence Immunoassay (ECLI) detection technique based on a ruthenium derivate. The detection limit was 0.8 IU/L, and the limit of quantification (CV < 20%) was 1.1 IU/L. Concentrations of TRAb <1.2 IU/L were considered negative, concentrations of 1.2–1.7 IU/L were considered gray zone, and concentrations of ≥1.8 IU/L were considered positive for Graves’ disease. In 2023, intra-assay CV% was 5.2 at a concentration of 2.6 IU/L (27). The methods are different; thus, the exact TRAb levels of the two laboratories were not comparable.

### Statistical analysis

Statistical analyses were performed using SPSS©, version 28 for Windows. Comparisons between groups were performed using the Kruskal–Wallis and Mann–Whitney U-tests. Fischer’s exact test was used for dichotomous variables. Values are presented as median (interquartile range), mean ± standard deviation (SD), or numbers and percentages. A *p*-value of <0.05 was considered statistically significant.

## Results

### Basal characteristics

Altogether, serum concentrations of TRAb were analyzed in Gothenburg from 121 endometriosis patients. In total, 38 patients had isolated ovarian endometriosis, 82 patients had spread endometriosis, and information was missing in 1 patient. Of the 50 IBS patients included, 11 had IBS-C, 18 had IBS-D, 12 had IBS-M, and 4 had IBS-U. Five of the IBS patients suffered from weekly abdominal pain, but the association with altered bowel habits was lower than 30%. Therefore, they were classified as suffering from functional gastrointestinal disorder (FGID). Regarding TRAb analyses in Malmö, all 81 endometriosis patients included in the second cohort were analyzed, along with 50 IBS patients, of which 24 were the same as in Gothenburg. Of these 50 IBS patients, 9 had IBS-C, 17 had IBS-D, 14 had IBS-M, 3 had IBS-U, and 7 had FGID. The same controls were analyzed at both laboratories.

BMI differed between the groups, with the highest BMI in patients with endometriosis. No significant differences were identified between the groups regarding age. The prevalence of hypothyroidism and the use of levothyroxine was higher in endometriosis than in IBS and controls in both analysis cohorts (Table 1; Supplementary Table S1). Patients with endometriosis and IBS had many gastrointestinal symptoms, in contrast to the healthy controls (Supplementary Tables S2, S3).

**Table 1:** Basal characteristics for endometriosis patients, IBS patients, and controls analyzed in Gothenburg.

	Endometriosis *n* = 121	IBS *n* = 50	Controls *n* = 50	*P*-value
Age (years)	38 (33–44)	38 (31.5–41)	34.8 (29.1–42.1)	0.323
BMI (kg/m^2^)	24.7 (22.6–28.3)	23.5 (21.8–27.0)	22.2 (21.6–24.1)	0.001
Smoking (n, %)	13 (10.8)	4 (8.0)	2 (4.0)	0.574
Thyroid disease (n, %)				
Normal function	108 (89.3)	50 (100)	50 (100)	0.003
Hypothyroidism	12 (9.9)	0 (0)	0 (0)	0.005
Hyperthyroidism	1 (0.8)	0 (0)	0 (0)	0.662
Levothyroxine treatment (n, %)	10 (8.3)	0 (0)	0 (0)	0.013

IBS, irritable bowel syndrome. The Kruskal–Wallis test and Fischer’s exact test. Values are presented as median and interquartile range or numbers and percentages. A *p*-value of <0.05 was considered statistically significant.

### TRAb analyses

There was no significant difference in the number of patients with detectable serum TRAb between endometriosis (*n* = 10, 8.3%) and controls (*n* = 2, 4%) (*p* = 0.512) or between endometriosis and IBS (*n* = 3, 6%) (*p* = 0.758) with the method used in Gothenburg. There was no difference in the TRAb concentrations between endometriosis (0.26 (0.26–0.26) IU/L, 0.408 ± 0.982 IU/L) and controls (0.26 (0.26–0.26) IU/L, 0.268 ± 0.099 IU/L) (*p* = 0.260) or between endometriosis and IBS (0.26 (0.26–0.26) IU/L, 0.364 ± 0.537 IU/L) (*p* = 0.725). Seven of ten endometriosis patients with detectable TRAb had concentrations < 1.4 IU/L and three had concentrations > 1.8 IU/L (positive). The two controls with detectable TRAb had concentrations < 1.4 IU/L. One IBS patient had TRAb concentrations < 1.4 IU/L and two patients had concentrations > 1.8 IU/L. None of the detectable TRAb analyses were in the gray zone (Table 2).

**Table 2:** Participants with detectable TRAb in Gothenburg and/or Malmö.

Patients	Gothenburg				Malmö			
	<0.26	0.26–1.3 Negative	1.4–1.8 Gray zone	>1.8 Positive	<0.8	0.8–1.1 Negative	1.2–1.7 Gray zone	>1.7 Positive
Endo 1*				3.925				
Endo 2*		0.839						
Endo 3*				10.260				
Endo 4*		0.685						
Endo 5*		1.232						
Endo 6*		0.638						
Endo 7*		0.973						
Endo 8		0.671			<0.8			
Endo 9				1.882	<0.8			
Endo 10		0.496			<0.8			
Endo 11	<0.26					0.960		
Endo 12	<0.26					0.850		
Endo 13	<0.26					0.980		
Endo 14	<0.26					1.050		
IBS 1				3.665	<0.8			
IBS 2		0.905			<0.8			
IBS 3				1.895	<0.8			
IBS 4*						0.930		
IBS 5*						0.836		
IBS 6*								3.520
IBS 7	<0.26						1.290	
Control 1		0.510					1.560	
Control 2		0.905			<0.8			
Control 3	<0.26							2.080
Control 4	<0.26							2.330
Control 5	<0.26					0.830		

Endo, endometriosis, IBS, irritable bowel syndrome. The concentration of TSH receptor antibodies (TRAb) in serum analyzed in Gothenburg (26) and in Malmö (27), given in IU/L. * Were only analyzed in one of the laboratories.

Similar results were found when TRAb was analyzed in serum samples with the method used in Malmö. TRAb was not more commonly detected in endometriosis patients (*n* = 4, 4.9%) than in controls (*n* = 4, 8.0%) (*p* = 0.710) or IBS patients (*n* = 4, 8.0%) (*p* = 0.710). There was no significant difference in concentrations of TRAb between endometriosis (0.8 (0.8–0.8) IU/L, 0.808 ± 0.039 IU/L) and controls (0.8 (0.8–0.8) IU/L, 0.872 ± 0.296 IU/L) (*p* = 0.524) or between endometriosis and IBS (0.8 (0.8–0.8) IU/L, 0.868 ± 0.389 IU/L) (*p* = 0.585). Concentrations of TRAb >1.7 IU/L were considered positive, and concentrations 1.2–1.7 IU/L were considered gray zone values, according to the laboratory protocol in Malmö (27). All four endometriosis patients with detectable TRAb had concentrations < 1.2 IU/L. One control had concentrations < 1.2 IU/L, one was in the gray zone, and two had concentrations > 1.7 IU/L (positive). Two IBS patients had concentrations < 1.2 IU/L, one was in the gray zone, and one had concentrations > 1.7 IU/L (Table 2).

Of the ten endometriosis patients with detectable TRAb (≥0.26 IU/L) analyzed in Gothenburg, three were also analyzed in Malmö. However, two of them were under the detection limit of 0.8 IU/L in Malmö. The one that was positive and over 1.8 IU/L in Gothenburg was not detectable in Malmö. Of the four endometriosis patients with detectable TRAb analyzed in Malmö, all four were also analyzed in Gothenburg. However, none of them had detectable TRAb in Gothenburg. Of the three IBS patients with detectable TRAb analyzed in Gothenburg, all were also analyzed in Malmö. They were all above 0.8 IU/L in Gothenburg, and two were positive at a level of > 1.8 IU/L, but none was detectable in Malmö. Of the four IBS patients with detectable TRAb analyzed in Malmö, only one was also analyzed in Gothenburg but was not detectable. TRAb was detectable in two controls in Gothenburg, and it was detectable in four controls in Malmö. Of these, only one was detectable in both analyses (Table 2). Among the 13 endometriosis patients with thyroid disease, one with hypothyroidism had TRAb concentrations > 1.8 IU/L and the rest had concentrations < 0.26 IU/L (or – undetectable TRAb). Of 16 subjects with detectable TRAb at any of the two departments, seven had TRAb in the gray zone or were positive in only one of the laboratories (Table 2).

There was no difference in localization of endometriosis, age, BMI, smoking, or gastrointestinal symptoms between those endometriosis patients with or without detectable TRAb (data not shown).

## Discussion

The main finding of the present study was that the use of current clinical laboratory setups to analyze TRAb IgG cannot be used to discover elevated levels of the autoantibodies in endometriosis as previously described using other methods (10, 11).

The laboratory setup for clinical use of TRAb is designed to discover true thyroid disorders, with high specificity and sensitivity for thyroid disorders, since this is the goal for the clinicians when using the analysis (27). Therefore, the methods have been developed over the last years to be more specific for Graves’ disease (28). Regarding previous findings in endometriosis, the elevated levels of TRAb were above the detection limit but below or within the gray zone (10). Thus, the TRAb levels in endometriosis had a quite distinct pattern from the pattern in thyroid disease, where the presence of TRAb for diagnostic purposes demands levels above the definition for positive concentrations (27). The research question to find slightly elevated levels of TRAb below or within the gray zone in endometriosis will need another laboratory test and other detection limits. Lower levels of detection may be applied in endometriosis diagnosis than in thyroid diagnosis, where small elevations may also be of importance for use as a potential biomarker.

The previous findings of elevated TRAb concentrations in endometriosis by two different methods to analyze IgG and IgM autoantibodies, which could not be confirmed in the present study, raise some questions. The reactivity measured as TRAb in endometriosis may depend on quite another autoantibody. This was considered at our laboratory, which is why we analyzed several other autoantibodies against follicle-stimulating hormone (FSH), human chorionic gonadotropin (hCG), luteinizing hormone (LH), and their receptors, without finding any autoantibodies suggesting cross-reactivity (11). Furthermore, TSH receptors have been identified in the endometrium and the ectopic endometrial tissue (5–7). Theoretically, there may be different subclasses of TSH receptors in different organs, although no proof of this has been published. In addition, there may be heterogeneity among TRAb, and TRAb with different antigenic epitopes have been found in patients with autoimmune thyroid diseases (29, 30). There may also be slightly different TRAb in endometriosis compared with TRAb in thyroid disease.

Even if we could not identify TRAb as a biomarker of endometriosis in this study, it is important to evaluate thyroid function in women with gastrointestinal symptoms, since thyroid diseases are common in the population and may render similar gastrointestinal symptoms as IBS and endometriosis (10, 12). Furthermore, it has previously been described that thyroid disease is common in both endometriosis and IBS (31, 32). Gastrointestinal symptoms may render the patient the IBS diagnosis, although the subject possibly only suffers from thyroid disease. This introduces considerable inaccuracy in the diagnosis setting, with some diagnoses only set by symptom questionnaires (IBS) and some diagnoses often overlooked with inadequate clinical examinations (endometriosis and thyroid diseases) (20). On average, it takes several years to get a diagnosis of endometriosis (15).

There are several limitations of the present study. First, it is a rather small cohort analyzed. No power analysis was conducted, and the results could depend on the study being underpowered. The small positive group of patients with endometriosis makes it difficult to detect small differences between groups and impairs the implications of the robustness of the conclusions. Furthermore, the detection limit was not the same at both laboratories. We did not repeat the IgM analysis of TRAb, due to the fact that it is not a clinically available method. Our intention was to use established methods to identify biomarkers. However, it is difficult to identify stable biomarkers that can be used and repeated over years in daily clinical practice. The cross-sectional study design limits the assessment of temporal relationships and the ability to predict disease progression and treatment response. However, you can still use a biomarker to detect the presence or absence of the disease. Another limitation is that both the IBS patients and controls may suffer from undiagnosed endometriosis. Many patients with gastrointestinal symptoms are considered to suffer from IBS without any further clinical examinations.

## Conclusion

Current clinical routines to analyze TRAb could not reveal elevated presence or levels of TRAb autoantibodies in endometriosis in this exploratory study. However, TRAb may be used in a laboratory experimental setting for further research and characterization of the reactions observed in the older analyses. The role of TSH receptors and autoantibodies against TSH receptors in the pathophysiology of endometriosis deserves further research in larger cohorts.

## Data Availability

The raw data supporting the conclusions of this article will be made available by the authors, without undue reservation.
