# Supplementary material for: Elevated levels of TRAb IgG autoantibodies are not recognized in endometriosis by the current clinical methods
Source: Front Med (Lausanne). 2025 Sep 22;12:1612079. doi: 10.3389/fmed.2025.1612079 (PMC12497825; doi:10.3389/fmed.2025.1612079)
Supplement: Supplementary file 1 [file Table_1.DOCX]

Supplementary Material

**Supplementary Table S1.** Basal characteristics for endometriosis patients, IBS patients and controls analyzed in Malmö

|  | **Endometriosis n=81** | **IBS**  **n=50** | **Controls**  **n=50** | **P-value** |
| --- | --- | --- | --- | --- |
| **Age (years)** | 37 (33–44) | 35 (27.8–42) | 34.8 (29.1–42.1) | 0.365 |
| **BMI (kg/m^2^)** | 25.3 (22.8–28.9) | 24.6 (22.1–28.2) | 22.2 (20.6–24.1) | 0.001 |
| **Smoking (n, %)** | 12 (14.8) | 5 (10.0) | 2 (4.0) | 0.351 |
| **Thyroid disease (n, %)** |  |  |  |  |
| Normal function | 74 (91.4) | 49 (98.0) | 50 (100) | 0.041 |
| Hypothyroidism | 6 (7.4) | 1 (2.0) | 0 (0) | 0.022 |
| Hyperthyroidism | 1 (1.2) | 0 (0) | 0 (0) | 0.539 |
| **Levothyroxine treatment (n, %)** | 7 (8.6) | 1 (2.0) | 0 (0) | 0.041 |

IBS=irritable bowel syndrome. Kruskal-Wallis test and Fischer’s exact test. Values are presented as median and interquartile range or numbers and percentages. P <0.05 was considered statistically significant.

**Supplementary Table S2.** Gastrointestinal symptoms in endometriosis patients, IBS patients and controls analyzed in Gothenburg

|  | **Endometriosis n=121** | **IBS**  **n=50** | **Controls**  **n=50** | **P-values** |
| --- | --- | --- | --- | --- |
| Abdominal pain | 49 (14–76) | 50 (30–65) | 0 (0–0) | <0.001 |
| Diarrhea | 25 (2–51) | 51 (9–74) | 0 (0–3) | <0.001 |
| Constipation | 34 (2–64) | 56 (5–82) | 0 (0–16) | <0.001 |
| Bloating and flatulence | 57 (21–77) | 80 (62–90) | 3 (0–17) | <0.001 |
| Vomiting and nausea | 14 (2–52) | 13 (1–40) | 0 (0–5) | <0.001 |
| Intestinal symptom´s influence on daily life | 52 (14–80) | 75 (64–86) | 0 (0–8) | <0.001 |
| Psychological well-being | 44 (13–65) | 39 (17–64) | 17 (0–51) | 0.005 |

Symptoms assessed by the visual analog scale for irritable bowel syndrome (VAS-IBS) [23] on VAS scales ranging from 0 (absence of symptoms) to 100 mm (maximal symptoms). Values are given as median (interquartile ranges). Kruskal-Wallis test. P<0.05 was considered statistically significant.

**Supplementary Table S3**. Gastrointestinal symptoms in endometriosis patients, IBS patients and controls analyzed in Malmö

|  | **Endometriosis n=121** | **IBS**  **n=50** | **Controls**  **n=50** | **P-value** |
| --- | --- | --- | --- | --- |
| Abdominal pain | 48 (12–72) | 50 (37–70) | 0 (0–0) | <0.001 |
| Diarrhea | 24 (0–49) | 53 (11.5–77) | 0 (0–3) | <0.001 |
| Constipation | 41 (3–65) | 59 (8–75) | 0 (0–0) | <0.001 |
| Bloating and flatulence | 52 (21–77) | 80 (53–90) | 3 (0–17) | <0.001 |
| Vomiting and nausea | 12 (0–45.5) | 12 (3–37) | 0 (0–5) | <0.001 |
| Intestinal symptom´s influence on daily life | 49 (8–80) | 77 (65–87) | 0 (0–8) | <0.001 |
| Psychological well-being | 46 (15–65) | 50 (17–66) | 17 (0–51) | 0.006 |

IBS=irritable bowel syndrome. Symptoms assessed by the visual analog scale for irritable bowel syndrome (VAS-IBS) [23] on VAS scales ranging from 0 (absence of symptoms) to 100 mm (maximal symptoms). Values are given as median (interquartile ranges). Kruskal-Wallis test. P<0.05 was considered statistically significant.
